# The Prevalence of Intestinal Parasitic Infections in Ahvaz, Southwest of Iran, during 2007–2017

**Published:** 2019-11

**Authors:** Roya ALASVAND JAVADI, Forough KAZEMI, Somayeh FALLAHIZADEH, Reza ARJMAND

**Affiliations:** 1. Student Research Committee, Ahvaz Jundishapur University of Medical Sciences, Ahvaz, Iran; 2. Department of Parasitology, Faculty of Medicine, Ahvaz Jundishapur University of Medical Sciences, Ahvaz, Iran; 3. Health Research Institute, Infectious and Tropical Diseases Research Center, Ahvaz Jundishapur University of Medical Sciences, Ahvaz, Iran

**Keywords:** Prevalence, Intestinal parasitic infections, *Giardia lamblia*, *Blastocystis hominis*, Iran

## Abstract

**Background:**

Intestinal parasitic infections (IPIs) are frequently considered one of the public health problems worldwide. Therefore, we aimed to evaluate the prevalence of IPIs among patients.

**Methods:**

In this Retrospective cross-sectional study, 50000 stool samples (24551 males) were collected among males and females referred to Naft Hospital of Ahvaz, southern Iran during 2007–2017. At first, the collected specimens were macroscopically observed for the presence of trophozoites, eggs, cysts using the procedure of direct as well as the method of formalin-ether concentration.

**Results:**

Of 50000 samples, 2878 (5.75%) cases were positive for IPIs that 1426 and 1452 cases were observed in the males and females, respectively. *Giardia lamblia* with 887 (1.774%) cases, *Blastocystis hominis* with 784 (1.568%) cases, *Entamoeba histolytica/dispar* with 685 (1.37%) cases, *E. coli* with 357 (0.714%) cases, *Trichomonas hominis* with 43 (0.086%) cases, *Chilomastix mesnili* with 40 (0.08%) cases, *Hymenolepis nana* with 38 (0.076%) cases, *Lodamoeba butschlii* with 25 (0.05%) cases, *Endolimax nana* with 18 (0.036%) cases, *Taenia saginata* proglottid with one (0.002%) case were found.

**Conclusion:**

Our finding showed a relatively high prevalence of IPIs among people referred to Naft Hospital of Ahvaz, southern Iran during 2007–2017.

## Introduction

Intestinal parasitic infections (IPIs) are frequently considered one of the public health problems in worldwide (1). Annually, about 450 million males and females suffer from these infections as well as more than two hundred thousand deaths have been annually reported (1). Some factors related to intestinal parasitic infections are including illiteracy, the geographical location of the areas, cultural conditions, lack of health care, economic conditions, poverty, social conditions, tropical wet climate and safe drinking water (1–3).

In most cases, the infection is asymptomatic but in some cases, these parasites can lead to physical health disorders including vitamin and iron deficiencies, diarrhea, vomiting, and abdominal pains (3, 4) as well as can cause the risk of other infections, including tuberculosis, viral infections, and malaria (5, 6), in particular in immunodeficient people and children (7). In some different areas of Iran, a relatively high outbreak of the infections have been frequently reported (1, 4, 8, 9), especially in Khuzestan Province southwest of Iran, (1). Given the weather conditions of this city, the evaluation of the rate of the infection in Ahvaz City, southern Iran with tropical climates is essential; therefore, the aim of this study was to evaluate the prevalence of IPIs among individuals referred to Naft hospital of Ahvaz, Khuzestan Province, southwest of Iran, during 2007–2017.

## Methods

### Ethical aspects

All the steps of the study were confirmed by the Ethics Committee of Ahvaz Jundishapur University of Medical Sciences as well as informed consent was obtained from all males and females included in the research.

### Study population

The current research was a Retrospective cross-sectional study. Males and females referred to Naft hospital of Ahvaz, Khuzestan Province, southwest of Iran, during 2007–2017, were selected as the study population. Initially, the purpose of the research was conveyed to the study population and in the next stage, 50000 stool samples (24551 males and 25449 females) were collected among the individuals.

### Stool examination

The fecal specimens (1.5–2.5 gr) were obtained from the study population and placed in labeled plastic vials. Less than 60 min after collecting, the collected samples were examined immediately. In the next stage, the collected specimens were macroscopically observed for the presence of trophozoites, eggs, cysts using the procedure of direct as well as the method of formalin-ether concentration. In summary, one gram the fecal samples were carefully mixed with four-gram formal saline. After filtering, adding diethyl ether and centrifuging, the obtained sediment was stained with 0.85% iodine and microscopically examined by light microscope at a magnification of 100X (10).

## Results

Table 1 shows the prevalence and microscopic identification of IPIs among subjects. Of 50000 collected specimens, 2878 (5.75%) cases were be positive for IPIs that 1426 and 1452 cases were observed in the males and females, respectively. The highest prevalence was related to *Giardia lamblia* (*G. lamblia)*, *Blastocystis hominis* (*B. hominis*) and *Entamoeba histolytica/dispar* (*E. histolytica/dispar*) with 887 (1.774%), 784 (1.568%) and 685 (1.37%) positive samples, respectively. Moreover, other IPIs were detected in the study that mentioned in Table 1.

**Table 1:** The prevalence and microscopic identification of IPIs among males and females referred to Naft Hospital of Ahvaz, southwest of Iran, during 2007–2017

***Parasite species***	***Frequency (n)***	***Percentage (%)***	***Male (n)***	***Female (n)***
No. of samples (n=50000)				
*Trichomonas hominis*	43	0.086	20	23
*Giardia lamblia*	887	1.774	447	440
*Entamoeba coli*	357	0.714	174	183
*Entamoeba histolytica/dispar*	685	1.37	345	340
*Blastocystis hominis*	784	1.568	387	397
*Lodamoeba butschlii*	25	0.05	13	12
*Chilomastix mesnili*	40	0.08	16	24
*Endolimax nana*	18	0.036	8	10
*Hymenolepis nana*	38	0.076	16	22
*Taenia saginata proglottid*	1	0.002	0	1
Total	2878	5.75	1426	1452

## Discussion

Intestinal parasitic infections (with a prevalence of 30%–60%) are causing important problems in both public and individual health (11). Based on our findings, the overall outbreak rate of IPIs was 5.75%. This finding was consistent with conducted investigations in Karaj (center of Iran) and Qazvin cities (north of Iran) with the overall prevalence of 4.7% (12), 5.8% (2), respectively. On the other hand, a higher outbreak rate of IPIs was reported in Mazandaran province (north of Iran), as well as Hamadan (west of Iran) and Isfahan (center of Iran) cities with the overall prevalence of 9.1% (13), 35.1% (14), 10.42% (15), respectively.

*G. lamblia* is the most frequent protozoa that can lead to diarrhea (1). The results of our study showed that the highest prevalence for intestinal parasitic infections in Ahvaz was related to *G. lamblia* with 887 (1.774%) positive samples. While in some studies, *B. hominis* (15) in Isfahan*,* and/or *E. coli* (2) in Qazvin were found as the most common. However, our finding with a prevalence of 887 (1.774%) cases for the protozoa in Ahvaz was lower than Kashan City (2.7%) (16), as well as Karaj City (3.8%) (12). In addition, other results of the study indicated that *B. hominis* with an outbreak of 784 (1.568%) cases was observed as the second common parasite obtained from individuals referred to Naft hospital of Ahvaz. While in the study in Isfahan (15), *B. hominis* was detected as the most common parasite.

On the other hand, *E. histolitica/dispar* is the second common parasite causing death in people infected with parasitic diseases (17). In the study, *E. histolitica/dispar* was found as the third common parasite in persons referred to Naft hospital of Ahvaz with a prevalence of 685 (1.37%) cases that the prevalence rate was consistent with investigation in Iran during 1988–2009 (18). It was showed that of 234,570 specimens, 1.3% were infected with the parasitic infection.

## Conclusion

Our finding showed a relatively high prevalence of IPIs among people referred to Naft Hospital of Ahvaz, southern Iran during 2007–2017.

## Ethical considerations

Ethical issues (Including plagiarism, informed consent, misconduct, data fabrication and/or falsification, double publication and/or submission, redundancy, etc.) have been completely observed by the authors.
